# INFLUENCE OF BREASTFEEDING ON CONSUMPTION OF SWEETENED BEVERAGES OR FOODS

**DOI:** 10.1590/1984-0462/;2018;36;2;00008

**Published:** 2018-01-08

**Authors:** Adriana Passanha, Maria Helena D’Aquino Benício, Sonia Isoyama Venâncio

**Affiliations:** a Universidade de São Paulo, São Paulo, SP, Brasil.; b Secretaria Estadual da Saúde de São Paulo, São Paulo, SP, Brasil.

**Keywords:** Food consumption, Breast feeding, Infant, Public health, Consumo de alimentos, Aleitamento materno, Lactente, Saúde pública.

## Abstract

**Objective:**

To verify whether breastfeeding is associated with lower prevalence of consumption of sweetened beverages or foods in infants.

**Methods:**

This is a cross-sectional study with data collected from the Survey on Prevalence of Breastfeeding conducted in Brazilian municipalities in 2008. A representative sample of 14,326 infants aged 6 to 11.9 months of age, residents of 75 municipalities in the State of São Paulo, Southeastern Brazil, was studied. The influence of breastfeeding on the consumption of sweetened beverages or food products was analyzed by multilevel Poisson regression. Variables with *p*<0.20 in the crude analysis were included in the multilevel analysis.

**Results:**

Most infants were on breastfeeding (56.1%). The prevalence of sweetened drinks or foods consumption was 53.3%. The consumption of sweetened products was shown to be less prevalent among breastfed infants after adjustment for confounding factors (PR 0.87; 95%CI 0.83-0.91).

**Conclusions:**

Breastfeeding was associated with lower consumption of sweetened beverages or foods. As an additional effect of actions aimed at promoting breastfeeding, a decrease in intake of sweetened products is expected among infants.

## RESUMO

**Objetivo:**

Verificar se o aleitamento materno associa-se a menores prevalências de consumo de bebidas ou alimentos adoçados entre lactentes.

**Métodos:**

Trata-se de um estudo transversal realizado com dados da Pesquisa de Prevalência de Aleitamento Materno em Municípios Brasileiros de 2008. Foi estudada uma amostra representativa de 14.326 lactentes, de 6 a 11,9 meses, residentes em 75 municípios do estado de São Paulo. Avaliou-se a influência do aleitamento materno sobre o consumo de bebidas ou alimentos adoçados mediante regressão de Poisson multinível. Permaneceram como variáveis de ajuste aquelas que apresentaram *p*<0,20 na análise bruta.

**Resultados:**

A maioria dos lactentes encontrava-se em aleitamento materno (56,1%), e 53,3% consumiram bebidas ou alimentos adoçados. O consumo de tais bebidas ou alimentos foi menos prevalente entre os lactentes amamentados, após ajustes pelas variáveis de confusão (RP 0,87; IC95% 0,83-0,91).

**Conclusões:**

A presença do aleitamento materno associou-se a menores prevalências de consumo de bebidas ou alimentos adoçados. Assim, pode-se esperar, como efeito adicional das ações de promoção ao aleitamento materno, que haja diminuição do consumo de bebidas ou alimentos adoçados entre lactentes.

## INTRODUCTION

Nutritional deficiencies or inadequate feeding in the first two years of life may lead to immediate impairments in the child’s health - increasing infant morbidity and mortality - and leave severe future sequelae, including higher prevalence of overweight and development of chronic noncommunicable diseases.^1^ The nutritional needs of an infant are met by exclusive breastfeeding up to six months of life; from that age on, other foods should be introduced in their diet while maintaining breastfeeding up to two years of age or more,^2^ since breast milk continues to nourish and protect them against diseases.^1^ ^,^ ^3^

It is important that healthy eating promotion include actions aimed at the formation of healthy eating habits from childhood, since foods offered in the first years of life become part of an individual’s lifetime habit.^1^ ^,^ ^3^ ^,^ ^4^ ^,^ ^5^ ^,^ ^6^ ^,^ ^7^Children are born with preference to sweet taste and learn to like food they are offered more frequently; thus, offering sweetened beverages and foods causes the child to lose interest in healthy options.^3^ ^,^ ^4^ ^,^ ^6^

Few national studies have addressed the introduction of foods of low nutrient density and high energy density in the first year of an infant’s life.^8^ Also, knowing the profile of complementary food introduction and the factors that influence the early introduction of unhealthy products is key for healthy eating habits promotion, including continued breastfeeding and prevention of high sugar intake and, therefore, obesity.^9^ Thus, the objective of this study was to verify whether breastfeeding is associated with lower prevalence of consumption of sweetened beverages or foods in infants.

## METHOD

Cross-sectional study based on information from municipalities of São Paulo that participated in the Maternal Lactation Prevalence Survey (*Pesquisa de Prevalência de Aleitamento Materno - PPAM*, acronym in Brazilian Portuguese) and conducted during the second phase of the National Polio Vaccination Campaign 2008. The PPAM was carried out by the Ministry of Health and aimed to pinpoint the situation of breastfeeding and complementary feeding in Brazil.^10^ The methodological aspects of PPAM were based on the Breastfeeding and Municipalities Project (*Projeto Amamentação e Municípios - AMAMUNIC*, acronym in Brazilian Portuguese), implemented between 1998 and 2012 in most municipalities of the State of São Paulo during the Vaccination Campaign. In order to select the sample in municipalities where more than 4,000 children were vaccinated, we used conglomerates in two stages: in the first, vaccination stations were drawn, and, in the second, children were drawn at each of them. The sample is considered equiprobabilistic in each participating municipality, since all children had the same probability of being chosen to the sample: larger stations were more likely to be drawn in the first stage, and children from smaller stations were more likely to be drawn in the second stage. For small municipalities, all children under one year of age who attended the second phase of the Vaccination Campaign were included in the survey.^11^

**Figure 1:**
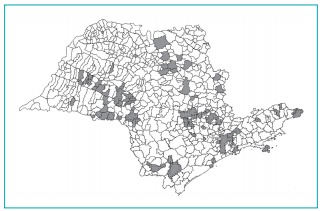
Distribution of the 75 São Paulo State municipalities included in the study.

In the present study, only infants aged 6 to 12 months incomplete were evaluated. We decided not to include children under six months of age because of the well-known recommendations of exclusive breastfeeding until so. Infants with no information about age or city of birth and municipalities with only one infant in the age range desired were excluded in view of the statistical analysis used. In 2008, in the State of São Paulo, 31,528 infants under one year of age were residents of 77 municipalities, of which 14,573 were in the age range of 6 to 11.9 months; of these, 246 were excluded for not meeting the predefined eligibility criteria. Two municipalities were excluded from the study: one for failing to meet the inclusion criteria and the other for having included only children under six months. Thus, 14,326 infants aged 6 to 11.9 months, living in 75 municipalities of Sao Paulo State participated in the study. The distribution of municipalities included in our sample can be seen in Figure 1.

São Paulo, one of the 27 federative units in Brazil, is located in its Southeastern region and, in 2015, had over 43 million inhabitants.^12^ ^,^ ^13^ Considered the richest of all federative units, São Paulo is also among the States with a high Human Development Index (HDI)^13^ and lower infant mortality rates.^12^

The questionnaire applied included closed questions about characteristics of infants and their mothers, and about food consumption based on the day before the survey - emphasis to recommended use of “current status” to describe infant feeding practices in order to minimize possible bias related to respondents’ memory.^14^ Based on the indicator of minimum dietary diversity by the World Health Organization (WHO),^14^ we created a variable called “diversified diet”, considering concomitant consumption of items from four food groups: meat, beans, vegetables, and fruits.

The information sought after by the present study was about “consumption of beverages or sweetened foods”: among beverages, consumption of processed juice, boxed coconut water or soft drinks was considered; as to foods, consumption of products added with sugar, honey, molasses or sweetener; outcomes were classified as “yes” if the infant had consumed any sweetened beverage or food. The study factor corresponded to breastfeeding, and infants receiving breast milk independently of other foods were considered on breastfeeding.^14^ The covariables of interest were the characteristics of infants (age: in full days, sex: male, female; birth weight: <2,500 g, ≥2,500 g, outpatient follow-up site: from private or public network, diversified diet: no, yes) and mothers (educational level in years of study: ≥12, 9-12, ≤8; age: ≥35 years, 20-35 years, <20 years, work situation: have a job, does not have a job; parity: primiparous, multiparous).

Regarding the variable of work for mothers, the category “on maternity leave” was considered as “missing”, since infants with more than six months of age would hardly have mothers in this condition. In total, 115 (1.0%) infants had ­mothers on maternity leave; assigning them “missing” status in the database did not cause significant differences to the sample (data not shown).

The association between independent variables and the variable response was assessed by crude analysis using Poisson regression, adjusted only to infant age. The individualized effect of study factor on the outcome was assessed by multilevel Poisson regression. Multilevel analysis was used according to the hierarchical organization of infant population in relation to their characteristics and their mothers’ characteristics (Level 1) in each municipality (Level 2), and to existence of intragroup correlation. Poisson regression was used to produce good point and interval estimates for Prevalence Ratio (PR) and because it is one of the best alternatives for cross-sectional studies with binary outcomes.^15^ PR values and respective 95% confidence intervals (95%CI) are also pointed out.

One must underline that PPAM is a research conducted with complex probabilistic sampling and therefore requires specific procedures when analyzed. Because of population differences across municipalities, each plan corresponds to a different sampling fraction represented by the estimated sample size versus number of children to be vaccinated. The inverse of this fraction was applied as infants’ weight in each municipality.^16^

Data were analyzed in the program Stata/SE 14.1. In the final model, variables that remained as adjustment factors were those with p<0.20 in the crude analysis. Covariables with more than two categories were introduced in the “dummy” model. Variables with PR between 0 and 1 were interpreted as factors reducing outcome prevalence, and PR>1 were interpreted as factors increasing outcome prevalence. The association between study factor and outcome was considered significant when p<0.05. Evaluation of multilevel model adjustment quality was verified by -2loglikelihood. The fixed effects/random intercept model was used, as described by Snijders and Bosker.^17^

This research project was approved by the Ethics Committee of the Public Health School (opinion 804543, year 2014). The mothers gave their verbal consent for questionnaire application.

## RESULTS

Table 1 shows the characterization of the studied population, the proportion of consumption of sweetened foods and beverages according to each feature and the results of the crude analysis. Approximately 3/4 of mothers aged 20 to 35 years. More than half had 9 to 12 years of schooling (52.4%), and most of them did not have a job (67.3%). Outpatient follow-up was made in public-network clinics in 61.4% of cases. Only 43.1% of infants were reported to have a diversified diet.

**Table 1:** Proportion of infants who consume sweetened beverages or foods^‡^ and respective Crude Prevalence Ratios according to characteristics of both infants and mothers.

Variable	n	%	Percentage of consumption^**‡**^	PR^**§**^	95%CI	p-value
Maternal schooling (years of study)						
≥12	1,733	15.0	43.1	1.00		<0.001^¶^
9|-12	6,057	52.4	53.2	1.23	1.16-1.32	
≤8	3,767	32.6	57.7	1.33	1.24-1.44	
Maternal age (years)						
≥35	1,438	12.3	48.5	1.00		<0.001^¶^
20|-35	8,754	74.8	53.0	1.09	1.01-1.18	
<20	1,508	12.9	59.9	1.26	1.16-1.37	
Parity						
Primiparous	5,924	51.0	52.4	1.00		0.036
Multiparous	5,690	49.0	54.4	1.04	1.00-1.07	
Work situation						
Has a job	3,722	32.7	51.1	1.00		0.002
Does not have a job	7,663	67.3	54.8	1.08	1.03-1.13	
Sex						
Male	7,200	50.3	54.8	1.00		0.443
Female	7,126	49.7	53.9	0.99	0.96-1.02	
Birth weight (grams)						
<2,500	1,232	9.2	53.2	1.00		0.917^¶^
≥2,500	12,189	90.8	53.2	1.00	0.91-1.10	
Outpatient care						
Private	5,310	38.6	49.0	1.00		<0.001
Public	8,432	61.4	57.8	1.17	1.12-1.23	
Diversified diet^†^						
No	8,099	56.9	50.1	1.00		<0.001
Yes	6,169	43.1	60.4	1.14	1.09-1.20	
Breastfeeding						
No	6,227	43.9	59.3	1.00		<0.001
Yes	7,955	56.1	50.4	0.85	0.81-0.89	

^‡^Consumption of soft drinks, industrialized juice, boxed coconut water or sweetened foods (with sugar, honey, molasses or sweetener). ^§^Prevalence Ratio (PR) values adjusted for infant age. ^†^Consumption of four food groups: meat, beans, vegetables, and fruit. ^¶^linear trend for *p*. Values of *p*<0,20 in bold.

The majority of infants were breastfed (56.1%). Regarding age, infants aged 6 to 8.9 months had a higher rate of breastfeeding compared to those aged 9 to 11.9 months (61.4 versus 50.6%). The prevalence of consumption of sweetened beverages or foods was 53.3%; 15.8% of the infants consumed processed juice/boxed coconut water, 10.9% soft drinks, and 43.1% consumed foods sweetened with sugar, honey, molasses or sweetener.

The lower the educational level or age range of mothers, the higher the consumption of sweetened beverages or foods. The consumption of sweetened beverages or foods was more frequent among infants of multiparous women or mothers who did not had a job, among those who had undergone outpatient follow-up in public health services, and among those with a diversified diet. In crude analysis, infants who were breastfed consumed less this type of food or beverage (Table 1).

Table 2 shows the results of multilevel analysis. The consumption of sweetened beverages or foods was shown to be less prevalent among breastfed infants after adjustment for confounding variables (PR = 0.87, 95%CI 0.83-0.91).

**Table 2:** Multilevel analysis: adjusted prevalence ratios and correspondent confidence intervals for consumption of sweetened beverages or foods^‡^, according to history of breastfeeding.

	Infants
Fixed effect - Constant	0.19 (0.16-0.21)
Breastfeeding	
No	1.00
Yes	0.87 (0.83-0.91)

Random effect - Constant: 5,11e-35 (1,15e-35-2,28e-34); -2 log likelihood: 8674,6714. ‡Consumption of soft drinks, industrialized juice, boxed coconut water or sweetened foods (with sugar, honey, molasses or sweetener). Prevalence Ratio (PR) values adjusted for infant age, maternal educational level, maternal age, parity, work situation, outpatient follow-up and diversified diet.

## DISCUSSION

In the present study, infants between 6 and 12 months of age who were breastfed were reported to consume less sweetened beverages or foods.

Limitations of the study include its cross-sectional design, which does not allow the establishment of causal relationships, and the fact that the feeding information would refer only to the day before the survey, which makes it impossible to assess how often sweetened products were consumed. On the other hand, one of the innovations posed was to explore the maintenance of breastfeeding as a protective factor for the formation of healthy eating habits in childhood. In addition, the multilevel analysis allowed to obtain estimates that take into account the hierarchical level of data and intragroup correlation. Also worth noting that PPAM is the most recent epidemiological study representing this population conducted in Brazil, meaning it is the most up-to-date database to evaluate infant feeding.

Data from the Food and Nutrition Surveillance System (*Sistema de Vigilância Alimentar e Nutricional - SISVAN*) show that, in 2008, the prevalence of overweight risk among children under two years in the State of São Paulo was 19.0%, and overweight and obesity were 6.7 and 7.2%, respectively. In 2015, no decrease was seen in such numbers, with rates of 18.9, 6.6 and 7.2% for overweight risk, overweight and obesity, respectively.^18^ Such findings are worth of attention, since they include almost one third of infants in São Paulo followed up by SISVAN and possibly reflect inadequacies in their feeding habits. Although SISVAN coverage in the State of São Paulo is generally low, the information obtained from the nutritional monitoring can support political decision-making and help to plan, monitor, and manage programs aimed at the improvement of feeding and nutritional status of the population.^19^

Among infants from São Paulo, breastfeeding rate was 56.1%; among those aged 9 to 11.9 months, it was 50.6%. The II PPAM conducted in the Brazilian Capitals and Federal District, in 2008, found a higher prevalence of breastfeeding among Brazilian infants aging 9 to 11.9 months (58.7%) and showed the worst prevalence for this age group in São Paulo, compared to all capitals of the Southeast region: 48.8%.^16^ Although more than half of the infants is breastfed, the prevalence found for the state of São Paulo shows that most children had not receive breastmilk the day before. This is worrying, considering that, after six months of age, it remains an important source of calories and nutrients, also being a factor of protection against diseases.^1^

The prevalence of consumption of sweetened beverages or foods was high among infants in São Paulo (53.3%), and 15.8% of them consumed processed juice or boxed coconut water, and 10.9% would intake soft drinks. A study on the consumption of unhealthy foods based on data from II PPAM found lower rates compared to ours: among Brazilian infants from 6 to 11.9 months, 11.8% consumed processed juice or boxed coconut water, and 8.2% were usually offered soft drinks.^20^ Step 8 of the “Food guide for children under two years of age” determines that sugar, soft drinks, candies, and other goodies are to be avoided in the first year of life, as their consumption is harmful to a child’s health and often associated with anemia, overweight, and food allergies,^1^ as already shown by some studies.^7^ ^,^ ^21^ ^,^ ^22^

Regarding the influence of breastfeeding on the consumption of sweetened products, our findings corroborate those of previous studies. Hendricks et al.^23^ analyzed characteristics associated with eating practices among children aged 4 to 24 months and found that those who had been breastfed were less likely to consume sweetened items compared to others. Lande et al.^9^ evaluated factors associated with breastfeeding and food consumption in 12-month-olds and found that breastfed infants would intake less sweetened and sugar-added beverages. Saldiva et al.^20^ showed that, among infants less than 12 months of age, absence of breastfeeding was related to significantly higher consumption of sweetened foods.

Studies also point out the long-term effect of breastfeeding on early childhood eating habits. Park et al.^24^ stated that, at six years of age, children who had been breastfed for six months or more had significantly lower rates of consumption of sweetened products more than once a day. The authors further identified a greater chance of children consuming such beverages more than once a week at six years of age when these beverages were introduced in the second semester of their life. Another study comparing length of breastfeeding and dietary patterns at six years showed that the longer the breastfeeding, the lower the consumption of sweetened beverages.^25^

It is worth noting that the consumption of sweetened beverages or foods was more prevalent among infants from São Paulo with diversified diet. This can be attributed to diets with greater variety of foods, in which children are also offered a greater supply of sugar-rich beverages or foods. Despite having consumed healthy food the previous day, such as meat, beans, vegetables and fruit, these infants also consumed sweetened beverages or foods, which is worrying, as several studies have pointed out the harmful effects of such products in the early years of life, including increased prevalence of overweight and obesity,^7^ ^.^ ^26^ dental cavities^26^ ^,^ ^27^ and preference for sweet taste.^6^ A possible explanation for the high consumption of sugar-rich foods and beverages by infants is that sweet foods are often considered to satisfy their palate and leave them well nourished. It is likely that the early provision of such foods occurs as a result of lack of information for mothers and caregivers about the appropriate age for their introduction. Considering that infants do not have the autonomy to make their food choices and depend on what they are offered, proper orientation of mothers and caregivers as to healthy feeding is of supreme importance.

Mothers who choose to continue breastfeeding their children are more likely to encourage the consumption of healthy foods and to limit the consumption of those considered less healthy compared to non-breastfeeding mothers.^25^ It can also be argued that mothers who are oriented to the importance of breastfeeding maintenance up to two years of age or more have also been targeted as to foods not recommended in the first year of life. As shown in another study,^28^ nutritional counseling on breastfeeding and complementary feeding promotes positive changes in infant food intake. In this sense, the relevance of materials about infant feeding that are easily accessible and understood by health professionals for orientation of the population, such as the “Food guide for children under two years” by the Ministry of Health.^1^ In addition, the inclusion of recommendations on healthy eating in the first year of life as part of nutrition education policies and programs can help reduce the consumption of sweetened beverages or foods among infants and avoid the continuity of their consumption later on their lives.^24^

It is important to stimulate the implementation of the Breastfeeding and Feeding Strategy in Brazil (*Estratégia Amamenta e Alimenta Brasil - EAAB*), which has existed since 2012, in order to strengthen and encourage the promotion of breastfeeding and healthy food for children under two years of age within the scope of Public Health System (*Sistema Único de Saúde - SUS*), aiming to reduce practices that discourage breastfeeding and improve healthy complementary feeding in order to form healthy eating habits from childhood, to increase breastfeeding in children up to two years of age or more, and to reduce the prevalence of children who receive unhealthy and not recommended foods.^29^ It is also mandatory to foster the creation of policies and actions aimed at guiding mothers and caregivers about the importance of maintaining breastfeeding and restricting of foods not recommended during the first year of life, with the aim of improving the scenario of infant food consumption.

In the present study, breastfeeding was associated with lower consumption of sweetened beverages of foods among infants aging 6 to 12 months. Thus, as an additional effect of actions aimed at promoting breastfeeding, a decrease in intake of sweetened products is expected among infants.
